# Prediction-Based Energy Saving Mechanism in 3GPP NB-IoT Networks

**DOI:** 10.3390/s17092008

**Published:** 2017-09-01

**Authors:** Jinseong Lee, Jaiyong Lee

**Affiliations:** School of Electrical and Electronics Engineering, Yonsei University, 50, Yonsei-ro, Seodaemun-gu, Seoul 03722, Korea; jinseong.lee@yonsei.ac.kr

**Keywords:** uplink prediction, energy saving, NB-IoT

## Abstract

The current expansion of the Internet of things (IoT) demands improved communication platforms that support a wide area with low energy consumption. The 3rd Generation Partnership Project introduced narrowband IoT (NB-IoT) as IoT communication solutions. NB-IoT devices should be available for over 10 years without requiring a battery replacement. Thus, a low energy consumption is essential for the successful deployment of this technology. Given that a high amount of energy is consumed for radio transmission by the power amplifier, reducing the uplink transmission time is key to ensure a long lifespan of an IoT device. In this paper, we propose a prediction-based energy saving mechanism (PBESM) that is focused on enhanced uplink transmission. The mechanism consists of two parts: first, the network architecture that predicts the uplink packet occurrence through a deep packet inspection; second, an algorithm that predicts the processing delay and pre-assigns radio resources to enhance the scheduling request procedure. In this way, our mechanism reduces the number of random accesses and the energy consumed by radio transmission. Simulation results showed that the energy consumption using the proposed PBESM is reduced by up to 34% in comparison with that in the conventional NB-IoT method.

## 1. Introduction

Internet of things (IoT) is being applied in various fields, from small sensors to industrial control systems, by supporting the interconnection between devices, and it aims to improve the quality of life [1]. Recently, new challenges are emerging for machine-type communication (MTC), such as public safety and problems in the field of smart grids. In addition, it is predicted that the IoT market will rapidly grow above ten times that of cellular communications [2]. Furthermore, 5G technology, expected to be commercially available by 2020, includes the development of two IoT-specific features, namely, massive IoT, suitable for high-density networks, and mission-critical IoT, aimed to delay-sensitive services [3]. To support various services, IoT networks will coexist with different technologies according to the type of service, rather than being unified by a single solution.

The connectivity technology for IoT services is classified into two categories according to the coverage distance: short-range technology, for applications including smart home and smart health, and long-range technology, for applications including tracking and monitoring for connected cars and smart watches. Short-range connectivity relies on technologies such as wireless local area network (WLAN), Bluetooth, and ZigBee, and the resulting network connects devices based on multiple technologies and forms an infrastructure with a gateway to connect to external networks. In this architecture, however, network maintenance, operation cost, and network complexity rise exponentially as the number of devices grows. In contrast, long-range connectivity relies on cellular network technologies such as global system for mobile communications (GSM), wideband code-division multiple access (WCDMA), long-term evolution (LTE), and LTE-MTC that cover several kilometers and support both device mobility without connection loss and high-speed transmission. However, these technologies present drawbacks including protocol overhead, reduced battery life, and high cost.

**Figure 1:**
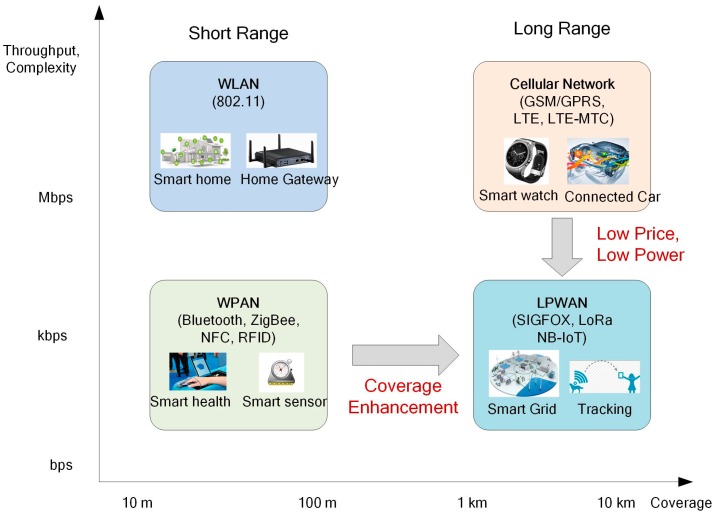
Internet of things (IoT) services according to coverage and throughput.

Some connectivity solutions currently require low-frequency operation and tolerate some amount of delay, but also demand to continuously operate for several years in a wide area [4,5]. However, the conventional connectivity technology cannot suitably satisfy these requirements. Given that the cellular network is optimized for voice, multimedia, and high-speed services, it is difficult to achieve low cost and low energy consumption for IoT modules. Likewise, the requirement of a gateway to provide connection with external networks in short-range connectivity technologies causes similar problems to those when using a wired or cellular network. To solve these problems, the low-power wide area network (LPWAN) technology was introduced. This technology extends the coverage area to several kilometers and improves the device cost and energy consumption by reducing the modem complexity. Figure 1 illustrates the LPWAN technology and IoT services according to coverage and throughput.

A LPWAN is designed with the following requirements: communication distance up to 40 km, thousands of devices supported by one base station, availability for over 10 years without battery replacement, and module price below US$5. Examples of LPWAN technologies that use the unlicensed industrial, scientific, and medical bands include SIGFOX and long range (LoRa), whereas the narrowband IoT (NB-IoT), recently introduced by the 3rd Generation Partnership Project (3GPP), uses the licensed bands [6]. The NB-IoT introduced the evolved packet system (EPS) optimization that reduces the protocol overhead of state transition to achieve low energy consumption in addition to reduce the modem complexity. In existing LTE networks, the control signals and application data are transmitted separately via the control and user planes, respectively, and a radio bearer setup is necessary to send application data. On the other hand, the NB-IoT enables a piggyback small application packet into the non-access stratum (NAS) signaling message. This scheme improves the transmission efficiency by skipping the bearer setup. Despite these features, there has been no progress in the scheduling request procedure to reduce energy consumption. Given that the NB-IoT is developed based on LTE, auxiliary procedures are necessary for the uplink radio resource allocation that is performed only by a random-access procedure.

In this article, we proposed a novel mechanism to improve energy consumption based on the predictive resource allocation. Prediction-based algorithms have been studied to guarantee the bandwidth and minimize the latency in poor channel condition. In [7], the authors investigated the predictability of user behavior to minimize bandwidth to achieve certain quality of service. However, this work does not exploit the non-continuous traffic characteristics and power consumption problem. In [8,9], the algorithm that predicts uplink radio resources by monitoring bandwidth measurement for the video stream was proposed. These papers analyzed real-time service which generates packet with short deterministic interval continuously. In contrast, our work is for IoT data traffic that only a few handshakes with small size packet exist. In [10], the authors made traffic model for machine-to-machine communication and proposed uplink packet prediction algorithm based on the idea that data traffic can be generated at similar time by machines in the same group with a high probability. But again, it does not exploit the correlation between uplink and downlink packet.

The remainder of this article is organized as follows. We briefly introduce the NB-IoT technology in Section 2 and identify the energy consumption problem caused by scheduling request procedures in Section 3. Then, we propose the algorithms to alleviate this problem in Section 4. Section 5 presents the simulation results and evaluation, and we provide a conclusion in Section 6.

## 2. NB-IoT

This section describes the NB-IoT key technologies to achieve low energy consumption and extended coverage, including baseband characteristics, data transmission scheme, extended discontinuous reception (eDRX), power saving mode (PSM), and control-plane cellular IoT (CP CIoT) EPS optimization.

### 2.1. NB-IoT with Other 3GPP Technologies

**Figure 2:**
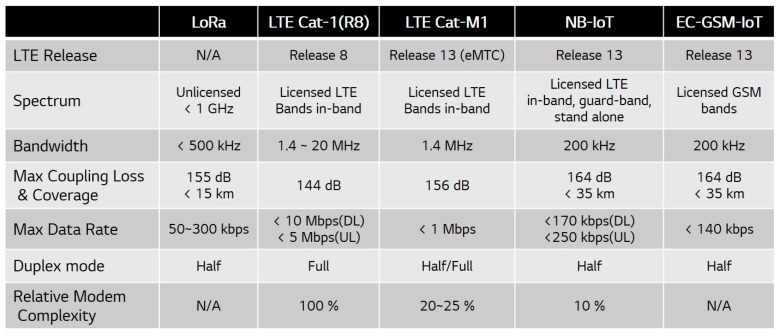
Comparison between narrowband (NB)-IoT and other technologies.

The 3GPP completed the first NB-IoT specification in 2016 and standardization is ongoing for schemes to strengthen mobility and reduce transmission delay. Although the NB-IoT appeared after LoRa and SIGFOX, it solves the congestion problem by using licensed bands, which enable more reliable services for mission-critical applications. In addition, given that the NB-IoT can coexist with GSM and LTE networks, which have already been successfully deployed, it is possible to use the existing network hardware and reduce the deployment cost [11]. Considering other 3GPP technologies, the maximum NB-IoT data rate has decreased, but its cell coverage is enhanced and the hardware complexity is reduced by 90% compared to that of LTE Cat-1, as shown in Figure 2. Hence, the NB-IoT can reduce the cost and energy consumption, which are the main shortcomings of cellular network technology for IoT devices. 

### 2.2. NB-IoT Key Technologies

#### 2.2.1. Baseband Characteristics

The radio frequency consists of a 180 kHz narrowband corresponding to one physical resource block (PRB) of an LTE network. Thus, it is possible to operate in the same band with an LTE network. In addition, half-duplex transmission, single antenna, single receiver chain, and low modulation scheme reduce the complexity and cost of the user equipment (UE). The downlink is composed of a 15 kHz subcarrier spacing using orthogonal frequency-division multiple access (FDMA), whereas the uplink can use both 15 kHz and 3.75 kHz single-carrier FDMA. Given the difficulty to increase the UE transmission power, the NB-IoT dedicates a 3.75 kHz subcarrier spacing for the uplink to support extended coverage.

#### 2.2.2. Deployment Scenario

**Figure 3:**
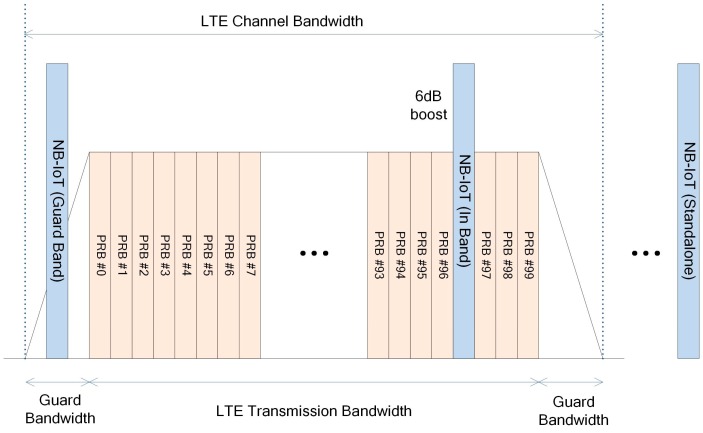
NB-IoT deployment scenarios.

The NB-IoT deployment is classified into in-band, standalone, and guard-band scenarios according to its relationship with the legacy cellular network as shown in Figure 3. The in-band scenario allocates 200 kHz of the LTE carrier to the NB-IoT and reuses the frequency that is already secured by the network operator. Hence, the deployment cost can be reduced because additional spectrum is not necessary. However, the resource map should be adjusted to guarantee the orthogonality with existing network and the available bandwidth for the LTE network can decrease. Next, the standalone scenario does not use LTE band resources, but assigns a dedicated spectrum to the NB-IoT. In addition, it can coexist with the GSM network by re-farming the network frequency. Finally, the guard-band scenario operates in the LTE guard band. Hence, it does not to require additional spectrum, but the number of available channels is limited. For instance, in the case of LTE with 20 MHz channel bandwidth, as shown in the Figure 3, only two 1 MHz guard bands are available, whereas the 10 MHz channel bandwidth has two 500 kHz guard bands [12,13].

#### 2.2.3. Coverage Extension

Although the LTE can operate up to 144 dB in the maximum coupling loss (MCL), the NB-IoT was designed to satisfy 164 dB MCL for extended cell size in environments with high penetration loss. An additional 20 dB link budget can be obtained from the lower bandwidths and repeating transmission. LTE operates in PRB units of 180 kHz, but the NB-IoT can operate with 15 kHz and 3.75 kHz, and thus can take additional 14 dB and 17 dB gains in terms of power spectral density, respectively. Likewise, the retransmission count of up to 128 for the uplink and 2048 for the downlink allows to obtain the additional link budget.

#### 2.2.4. Data Transmission Scheme

**Figure 4:**
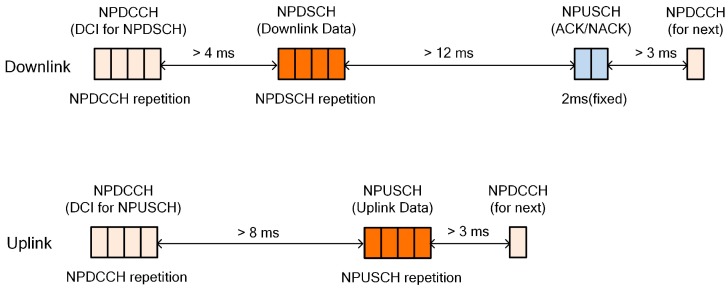
Time relation for data transmission.

Compared with LTE and for simplicity, the NB-IoT supports only one hybrid automatic repeat request (HARQ) that is asynchronous and adaptive for both uplink and downlink to support scheduling flexibility of the base station [14]. Figure 4 shows the physical channel and timing relation for uplink and downlink data transmission. A narrowband physical dedicated control channel (NPDCCH) carries the downlink control information (DCI) that includes scheduling delay, modulation coding scheme, and repetition number for both the narrowband physical uplink shared channel (NPUSCH) and narrowband physical downlink shared channel (NPDSCH). The NPUSCH conveys not only a medium access control (MAC) protocol data unit (PDU), but also HARQ acknowledgment (ACK)/negative acknowledgment (NACK) information, unlike LTE. The repetition number of each channel is determined from the base station based on the coverage-enhancement (CE) level of the UE, which is determined through the random-access procedure. In addition, the time interval between each channel can be distributed for the flexibility of scheduling policy. Since the NB-IoT is a half-duplex system, a transmission switching time above 3 ms is required.

#### 2.2.5. eDRX

Discontinuous reception (DRX) is a technology to deactivate the UE modem during a specific period that is negotiated with base station. Hence, the UE turns on the modem and monitors the downlink channel only during a specified time. Consequently, the transmission is delayed until the next monitoring period when the base station has a packet to send and the UE modem is deactivated.

**Figure 5:**
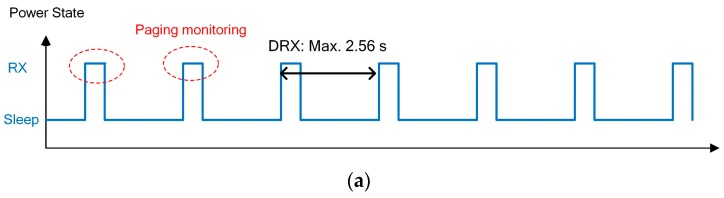

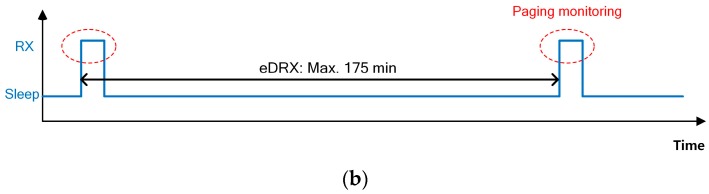
User equipment (UE) power state: (**a**) discontinuous reception (DRX); (**b**) extended discontinuous reception (eDRX).

DRX is classified into idle and connected DRX according to the radio resource control (RRC) connection state. In idle DRX, the RRC is not connected with base station, and the UE monitors only the paging signal for 1 ms per DRX period. Thus, it is possible to save energy by activating the modem only in the monitoring period. Moreover, as the DRX cycle becomes larger, the average energy consumption decreases and the battery life increases. However, a long DRX period increases the response time, thus compromising the user experience. Taking the user experience effects into account, the maximum DRX period of an LTE network is 2.56 s. In contrast, energy consumption is more important than an immediate response for the IoT. Thus, the DRX period can reach 175 min in the NB-IoT by employing hyper frame number. Figure 5 shows the maximum period of DRX and eDRX along with the UE power state.

#### 2.2.6. PSM

**Figure 6:**
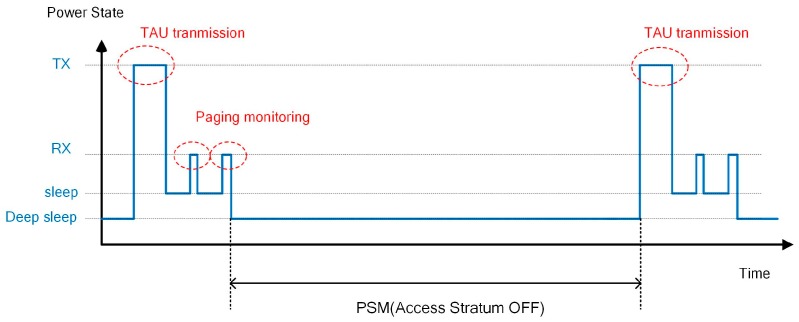
UE power state in power saving mode (PSM).

Some IoT devices do not require to frequently send packets and receive a packet from the network sporadically. For instance, in the case of a fire alarm, data transmission is mostly triggered from the temperature sensor, and it is not necessary to monitor paging except for firmware updates and keepalive. Thus, for some applications, the downlink latency is not important, and it is enough to monitor paging every several days. If paging does not need to be monitored for a long time, the UE is able to deactivate, besides the modem hardware, most parts of the device such as memory refresh. Figure 6 illustrates the relationship between the PSM operation and the UE power state. The PSM interval is negotiated and decided via a tracking area update (TAU) procedure with a maximum interval of several months.

#### 2.2.7. CP CIoT EPS Optimization

**Figure 7:**
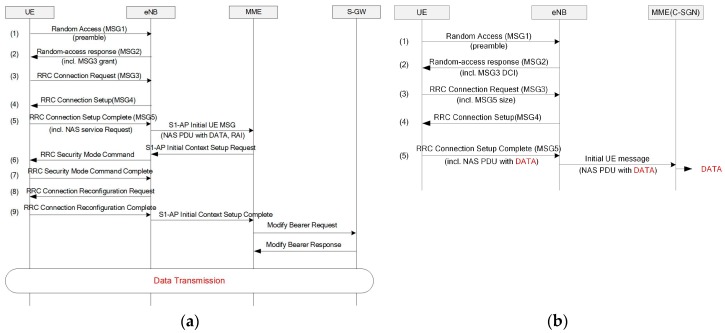
Packet transmission scheme: (**a**) existing long-term evolution (LTE); (**b**) NB-IoT with control-plane (CP) cellular IoT (CIoT) optimization.

The existing EPS is optimized for high-end services, including multimedia, voice, and Internet access. Thus, transmitting the IoT packet that is small and infrequent is inefficient through this system. Specifically, a connection setup is always required regardless of the packet size, and the EPS supports only IP packets with large overhead. Consequently, EPS optimization has been applied for the NB-IoT on both control and user plane aspects. In this paper, we focus on optimization for the control plane. The CP CIoT EPS optimization is designed to allow a piggyback application packet into the NAS PDU to reduce the radio resource usage when establishing the user-plane radio bearer. First, a CIoT-serving gateway node (C-SGN) that is a logical node and plays the role of mobility management entity (MME), a packet data network gateway (P-GW), and a serving gateway (S-GW) are used to reduce the network signaling overhead. In addition, encryption and header compression can be processed on the MME, and data transmission does not require the access stratum (AS) setup. Both AS security and user-plane radio bearer setup are mandatory in LTE networks. Thus, at least 9 messages are required before the transmission of the first application packet, as shown Figure 7a. On the other hand, the NB-IoT CP CIoT EPS optimization allows to transmit application packets after 4 messages, as shown in Figure 7b.

## 3. Problem Statement for Scheduling Request

Even after the radio bearer is established and the RRC is in the connected state (RRC_Connected), the uplink packets cannot be transmitted without a scheduling request procedure. In this section, we present the scheduling request procedure and the energy consumption problem in NB-IoT networks.

Similar to LTE networks, the NB-IoT radio resources for packet transmission are shared among UEs, and an evolved node B (eNB) scheduler dynamically assigns these resources to each UE based on the scheduling policy. Scheduling commands, which contain assigned time, resource, and decoding information, represent DCI transmitted via the NPDCCH. The UE decodes the NPDCCH information by using a radio network temporary identifier (RNTI) at specific times with the decoding period configured when the connection is established. This RNTI-based decoding identifies whether the radio resource for the NPUSCH is assigned to the UE.

**Figure 8:**
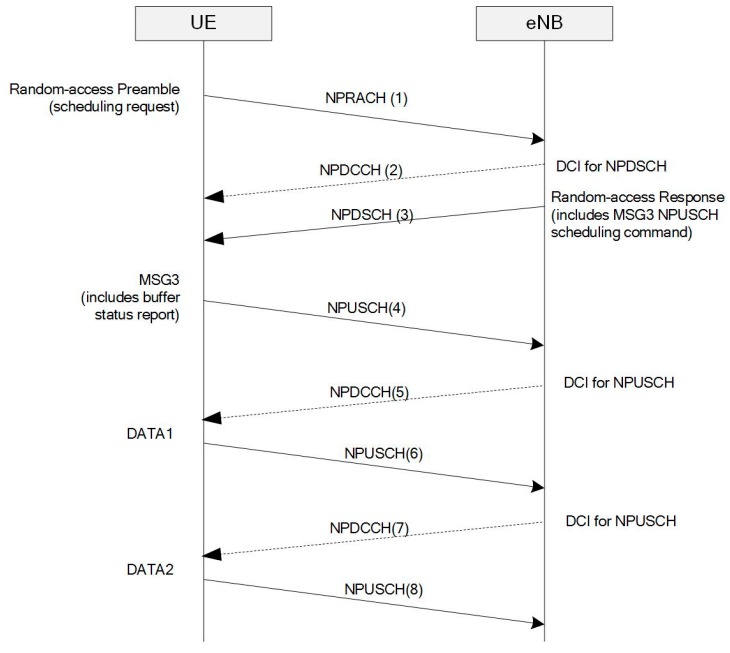
Scheduling request procedure with random access.

Figure 8 illustrates the uplink packet transmission procedure including the physical control channel in the case that the UE does not have any assigned radio resources for uplink. In contrast to LTE networks, the NB-IoT does not have dedicated radio resources for scheduling requests. Hence, the uplink radio resources can be only requested through a random-access procedure. Since this procedure competes with that of other devices, the contention resolution could fail and the UE retries random access after a back-off time. As shown in step (1) of Figure 8, the preamble is transmitted via a narrowband random-access channel (NPRACH). Then, the eNB sends the random-access response through the NPDSCH and NPDCCH. The NPDCCH includes the DCI to decode the NPDSCH, and the NPDSCH carries an identifier for contention resolution and a scheduling command for the next NPUSCH, as shown in steps (2) and (3) of Figure 8. The eNB scheduler has no information on the amount of data in the UE buffer. Hence, it first assigns a small radio resource to receive the UE buffer status information. Next, when the UE receives the uplink scheduling command, it sends the buffer status report through the scheduled NPUSCH, as shown in step (4) of Figure 8. Finally, the eNB acknowledges the UE buffer status and continuously assigns uplink radio resources until the data transfer corresponding to the reported size is completed, as illustrated in steps (5) though (8) in Figure 8.

Thus, although the radio bearer is established, additional interactions are required due to the scheduling request procedure. Given that the random-access procedure can be used to send a RRC connection request besides the scheduling request, the response message is typically composed of a 88-bit scheduling command, a size appropriate for that type of message [15]. Therefore, even packets with few bytes are difficult to transmit in MSG3 (step (4) of Figure 8), and another uplink transmission is required to complete the packet transmission, like step (6) of Figure 8. Given the NB-IoT repeating transmission for coverage enhancement, as the number of uplink application packets increases, so does the energy consumption.

**Figure 9:**
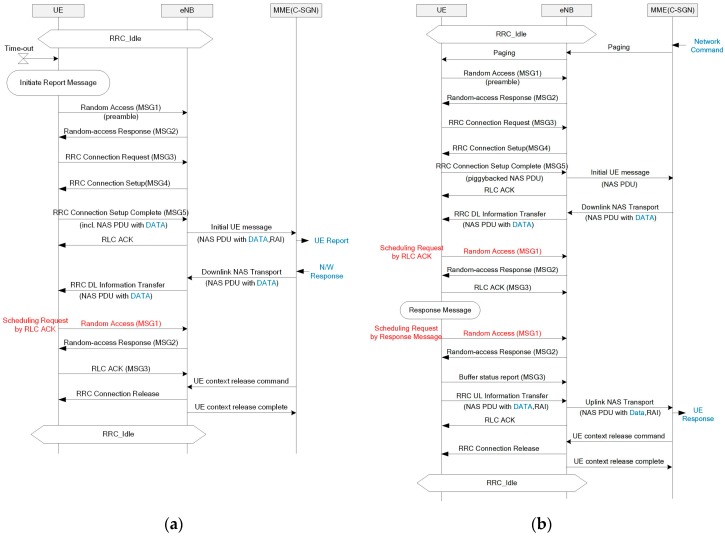
Handshake scenario: (**a**) UE report with network response; (**b**) network command with UE response.

Clearly, there is no effect of the scheduling request when a session completes after the transmission of one small packet, as that shown in Figure 7b. However, if a handshake between the UE and network is required, as shown in Figure 9, the battery consumption by the scheduling request increases. Figure 9a illustrates the scenario where the network server responds to the application report, and a radio link control (RLC) ACK packet corresponding to the downlink data should be transmitted with the scheduling request procedure. Figure 9b illustrates the scenario of a session triggered by the network command. In the worst case, two scheduling request procedures can occur by the RLC ACK and application response due to different processing times. As the active time increases by the random-access procedure, both the battery life and cell capacity can decrease due to failure on contention resolution.

**Figure 10:**
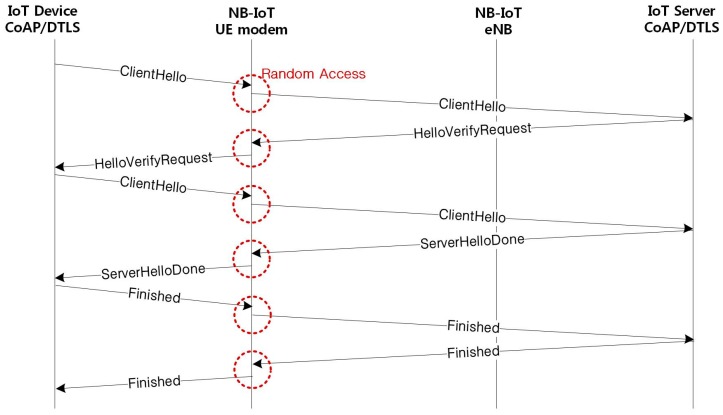
Datagram transport layer security (DTLS) 6-way handshake to create initial security context.

Besides the handshake triggered by application packets, the energy consumption problem arises by the transport protocol stack used in the IoT network, such as constrained application protocol (CoAP), datagram transport layer security (DTLS), and multicast domain name system (mDNS). The DTLS protocol, which is intensively investigated for the IoT [16], needs additional handshakes for security-context creation and resuming [17]. Given the memory cost for the server to maintain several security contexts from IoT devices, the refresh operation at the server is inevitable. In this case, an inappropriate timeout value can cause more scheduling request procedures and increase energy consumption. Figure 10 illustrates the initial 6-way handshake to create security context in the CoAP/DTLS protocol. The uplink DTLS and RLC ACK packets need a scheduling request procedure per transmission.

## 4. Prediction-Based Energy Saving Mechanism

In this section, we propose a prediction-based energy saving mechanism (PBESM) that allows predictive resource allocation to reduce the energy consumption caused by the scheduling request procedure in an NB-IoT network. The PBESM predicts the uplink occurrence and processing delay from packet inspection, and the eNB pre-assigns radio resources for uplink packet transmission. Thus, the PBESM allows to send uplink packets without requiring a scheduling request procedure.

### 4.1. Network Architecture with PBESM.

**Figure 11:**
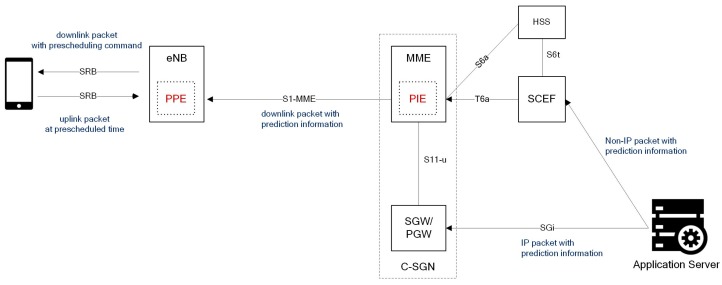
Network architecture with prediction-based energy saving mechanism (PBESM).

Figure 11 shows the NB-IoT network architecture that includes proposed mechanism. It is basically the same as the conventional network architecture and interface structure, but considers two new entities, namely, the packet inspection entity (PIE) and packet prediction entity (PPE). 

The PIE, which is logically located on the MME, determines the session type from the packet header inspection, e.g., protocol type, port number, and IP address. Then, the PIE predicts the occurrence of the uplink response message with strategy as o the paging delay in the PSM. 

Table 1. Moreover, it measures the response time for each session and sends this information to the PPE, which operates in the base station. The PIE is located on the MME because the paging delay in the PSM may pull the predictive processing delay in the wrong direction, and the MME can prevent this case. The estimated information is transmitted through the existing S1-MME interface in the downlink packets. Though the response message of the NAS and transport protocol (e.g., DTLS, CoAP, and transmission control protocol–TCP) can be predicted in the PIE, it is difficult to predict the application response due to message encryption and customized protocols. Therefore, the application server should support the prediction of uplink occurrence to profit from the PBESM. For prediction from the application server, the information about packet occurrence is trustable only due to the paging delay in the PSM. 

The PPE organizes the received uplink occurrence information and response time for each session. Using these data and the proposed algorithm, it predicts the processing delay, which is the time difference between the downlink transmission and uplink packet generation. Then, the eNB generates a prescheduling command that contains the uplink transmission time and modulation scheme, and the command is sent to the UE in a downlink packet via the NPDSCH. When the UE receives the command, it holds the uplink packet for the specified time without a scheduling request procedure.

#### Predictable Packet Type

There are five types of predictable packets, namely, RLC ACK, RRC and NAS signaling messages, transport layer response (e.g., TCP ACK and DTLS response), and application response. Table 1 lists the prediction entities and strategies for each packet type.

### 4.2. Prediction of Processing Delay

The processing delay for downlink packets varies according to the protocol category, application type, and device performance. The PPE manages the prediction metrics for each session and UE. We introduce the predictive processing delay, $T_{CID,PID}$ according to the cell ID (UE cell ID–CID) and the packet type (packet type ID–PID). Hence, we define the *k*-th predictive processing delay as $T_{CID,PID}\left(k\right)$ and modified predictive processing delay ${T^{\prime }}_{CID,PID}\left(k\right)$, which applies the carried information with weight (1−α) by

(1) $$T\prime_{CID,PID}\left(k\right)=\alpha \times \;T_{CID,PID}\left(k\right)+\left(1-\alpha \right)\times T_{info}$$

where $T_{info}$ is the received information from the MME.

For a given session and UE, the processing delay can be very similar with its previous value under the same UE load. Therefore, the proposed algorithm uses the previous value as a prediction reference. The prediction result and statistics of past predictions are also considered. Whether the previous prediction was successful can be confirmed through the decoding result of the preassigned radio resource. The variation of the predictive processing delay follows these criteria: if the previous prediction failed and the current probability, P, of successful delay prediction is smaller than the target probability, $P_{o}$, of successful delay prediction, then the PPE increases the predictive processing delay; if the previous prediction succeeded and P is greater than $P_{o}$, then the PPE reduces the predictive processing delay; whereas the PPE holds previous value otherwise. The corresponding expression is:

(2) $$T_{CID,PID}\left(i\right)=\left\{\begin{array}{ll} \min\left\{\left(1+\Gamma *R_{up}\right)\times {T^{\prime }}_{CID,PID}\left(i-1\right),\;T_{max}\right\}, & if\;P<P_{o}\;and\;CRC=NOK;\\ \max\left\{\left(1-\Gamma *R_{down}\right)\times {T^{\prime }}_{CID,PID}\left(i-1\right),\;T_{min}\right\}, & if\;P>P_{o}\;and\;CRC=OK;\\ {T^{\prime }}_{CID,PID}\left(i-1\right), & otherwise, \end{array}\right.$$

where:

- $\Gamma =\;\left|P-P_{o}\right|,$
- $R_{down}$ and $R_{up}$ are compensation steps,
- $R_{down}=R_{up}\times \left(1-P_{o}\right)/P_{o}$, and
- $T_{max}$ and $T_{min}$ are the upper and lower boundaries, respectively.

The variation step has a dependency on the target probability in (2). Hence, the predictive processing delay has a slower decrease compared with the rate of increase, as the target probability is larger. Γ has a role to reduce the variation step when the current probability of successful delay prediction is close to the target probability. Algorithm 1 shows the corresponding algorithm to predict the processing delay. The processing delay is estimated in lines 2 to 12, and the probability of successful delay prediction is updated in lines 13 to 23. Furthermore, a window calculation is applied to reduce the complexity and consider the most recent results, as described in lines 19 to 23. Initial values are chosen based on the simulation result.

**Algorithm 1.** Proposed prediction algorithm for processing delay in an NB-IoT network  1: **Initialization**: $P_{o}$=0.9, P=0.0, $R_{up}$=0.5, $R_{down}$= $\left(1-P_{o}\right)/P_{o}$, T(0)=0, α=0.8 for NAS/APP/IP packet, α=1.0 for RLC/RRC packet, CRC=0, k=1, $T_{max}$=30,000, $T_{min}$=10, $W_{p}$=256   2: **Prediction:**  3: k = k + 1  4: Γ = |P − $P_{o}$|  5: T = α
×
T(k−1) + (1 − α) × $T_{info}$
  6: **if**
P
$<P_{o}$ and CRC = 0 **then**  7:  T(k) = min{T
× (1 + $\Gamma \times R_{up}),\;T_{max}$}  8: **else if**
P
$>P_{o}$ and CRC = 1 **then**  9:  T(k) = max{T
× (1 −
$\Gamma \times R_{down}),\;T_{min}$}10: **else**11:  T(k) = T(k−1)12: **endif** 13: **NPUSCH result update:**14: **if** NPUSCH_CRC = OK **then**15:  CRC = 116: **else**17:  CRC = 018: **endif**19: **if**
k<
$W_{p}$
**then**20:  P = (P× (k-1) + CRC)/k
21: **else**22:  P = (P× ($W_{p}$-1) + CRC)/$W_{p}$23: **endif**

### 4.3. UE Procedure

Algorithm 2 describes the procedure to apply the PBESM in the UE side. The UE stores a prescheduling command, which is transmitted via NPDSCH, and checks the stored prescheduling command when the uplink packet occurred. If there is no pre-scheduling command, the UE follows the legacy scheduling request procedure. However, if there is stored information, the UE defers the uplink packet transmission until the prescheduled time without triggering the scheduling request procedure.

**Algorithm 2.** Proposed UE procedure in an NB-IoT network1: **Scheduling request procedure:**2: **if** scheduling request is triggered **then**3:  **if** scheduling command is already stored **then**4:    store uplink packet in the buffer.5:    delay uplink transmission by prescheduled time6:  **else**7:    process scheduling request procedure with RA8:  **endif**9: **endif**10: **Uplink Scheduling Procedure:**11: **if** TX time equals scheduling command time **then**12:  **if** buffer is not empty **then**13:    process NPUSCH transmission14:  **else**15:    ignore NPUSCH16:  **endif**17: **endif**


On the other hand, if the eNB fails to predict the processing delay and the UE does not have uplink packets to send at the prescheduled time, the UE does not send empty packets to save energy. In this case, the UE does nothing at the prescheduled time and follows the legacy scheduling request procedure when uplink packets are generated.

### 4.4. Uplink Procedure without Scheduling Request

**Figure 12:**
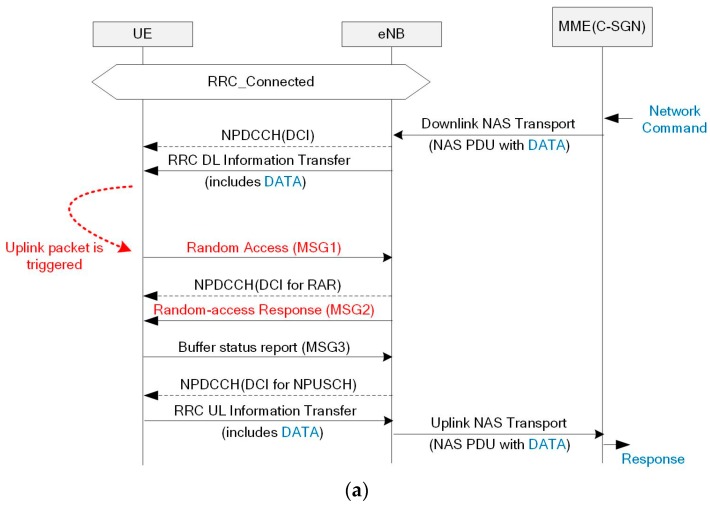

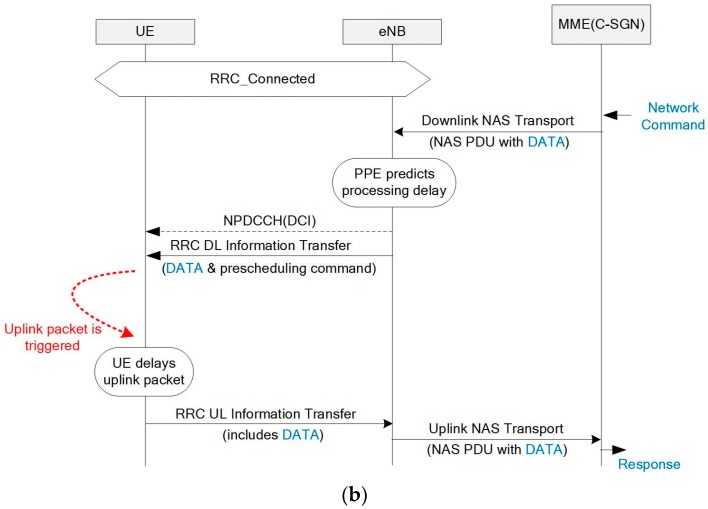
Network command and UE response scenario: (**a**) existing NB-IoT; (**b**) proposed PBESM.

Figure 12 compares the legacy NB-IoT and PBESM procedures for the scenario that the UE receives a network command and sends the response message. For the NB-IoT, the random-access procedure is performed to request uplink radio resources. However, the PBESM delays the uplink packet until the prescheduled time, and the random-access procedure is not executed. In view the UE TX operation, MSG1 and MSG3 procedures can be bypassed using proposed mechanism and battery consumption for message transmission can be substantially reduced. Furthermore, if the predictive processing delay is overestimated, the PBESM latency can be longer than that of the legacy NB-IoT procedure. However, this can be mitigated by setting the maximum predictive delay to a smaller value than the random-access procedure delay. Clearly, a large predictive processing delay improves the energy consumption for more sessions by reducing the prediction failure, but it causes more transmission latency. Hence, there is a tradeoff between latency and energy consumption.

## 5. Simulation and Results

This section describes the system-level simulation and corresponding results to validate the performance of the proposed PBESM for an NB-IoT network.

### 5.1. Simulation Setup

The parameters used to evaluate the proposed mechanism are listed in Table 2. The parameter names, values, and ranges follow the 3GPP specification [18]. The eNB scheduler is designed to select a small value to minimize the active time as much as possible. For the same reason, we assumed that the eNB and MME support release assistance indicator functionality, which means that the eNB can release the radio bearer immediately after the last packet transmission without monitoring the packet activity.

According to the 3GPP specification [19], cells classify the UEs into three coverage-enhancement levels depending on the radio condition. We classified the channel condition into good, medium, and bad conditions according to the MCL. Each channel condition is designed to have a 10 dB difference in MCL. The simulation metrics for each channel condition are listed in the Table 3.

#### 5.1.1. Simulation Scenario

We used five application-layer scenarios for performance evaluation as shown in Table 4. Case 1 corresponds to a UE report scenario, such as the periodic metering service [20], where the IoT device reports sensor values to the network using non-IP packets and receives an acknowledgment from the network to complete the session. In case 2, the application server checks the device status and sensor value. As in case 1, non-IP packets are used, but the session is triggered by a network command and finishes with the UE response and consequent network acknowledgment. Cases 3 to 5 have the same scenario as case 2 but employ IP. Cases 3 and 4 use CoAP/DTLS/UDP that is generally used in IP-based IoT systems [21]. Case 3 includes an initial context creation procedure for DTLS connection, and case 4 considers that the DTLS context already exists, but resuming is necessary. Finally, TCP is used in case 5, but it reduces the header size by robust header compression.

#### 5.1.2. Energy Consumption According to UE Power State

The power consumption according to the UE state was based on the value used in the 3GPP NB-IoT evaluation as listed in Table 5 [22]. The energy consumption is calculated using the power and the duration of each state.

#### 5.1.3. Processing Delay Distribution

The Pareto distribution was used for the processing delay to generate a downlink packet response. Scale parameter X m was 300 ms for the highest layer protocol over RRC and 80 ms for the other layers. We set tail index of the Pareto distribution between 2 and 4.

### 5.2. Simulator Evaluation

**Figure 13:**
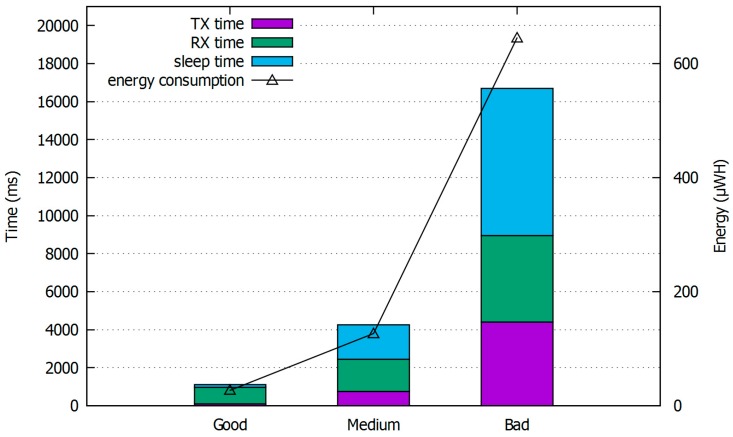
Active time and energy consumption for UE report scenario.

Figure 13 shows the simulation results of the non-IP UE report scenario that is used in the 3GPP NB-IoT evaluation to confirm the simulator performance. In this scenario, the application report is piggybacked to an *RRC connection setup complete* message, and the session is completed immediately after. The figure shows the energy consumption and active time at which the UE operates from its activation to the transmission completion according to the channel condition. As the channel quality improves and the session time reduces, the RX time is considerable in the total active time due to the acquisition of the master information block. This acquisition verifies the radio frame number after waking up from the deep-sleep state. Furthermore, as the channel quality reduces, both the active time and energy consumption increase. However, the growth rate for the energy consumption is larger than that of the active time, given the increase in the proportion of time in the TX state that consumes more energy than the other states.

### 5.3. Results

**Figure 14:**
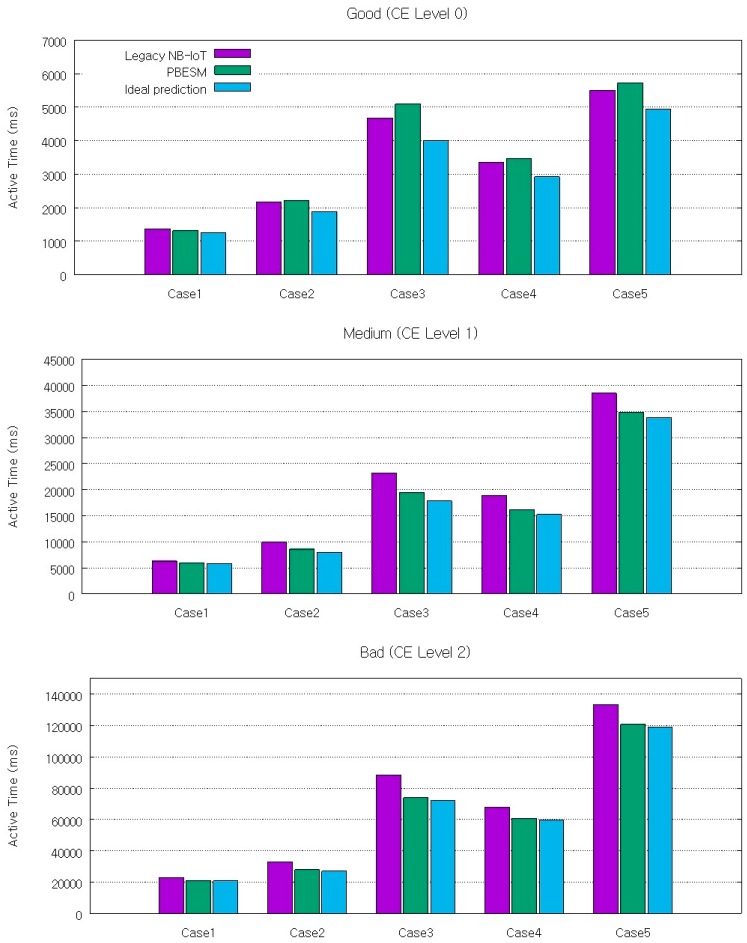
Active time according to channel condition for legacy NB-IoT, PBESM, and ideal prediction.

Figure 14 shows the active time for the case of legacy NB-IoT, PBESM, and ideal prediction. The ideal prediction is the case that applies the proposed mechanism, but it corresponds to a PPE prediction of processing delay with 0% error and it can be regarded as the improvement limit for the processing delay prediction. We considered a target probability of successful delay prediction of 90% and a tail index of 3 for the Pareto distribution. The ideal prediction can reduce the active time from 8% to 23% according to the channel condition and compared with the legacy NB-IoT. For the PBESM, the active time only reduces from 5% to 16% for the medium and bad channel quality. This performance difference is caused by the prediction error of the processing delay. In contrast, for a good channel quality, the active time rises by up to 8% depending on the scenario. The reason is that for a good channel quality, the time taken for random access is smaller than the prediction error of the proposed mechanism.

**Figure 15:**
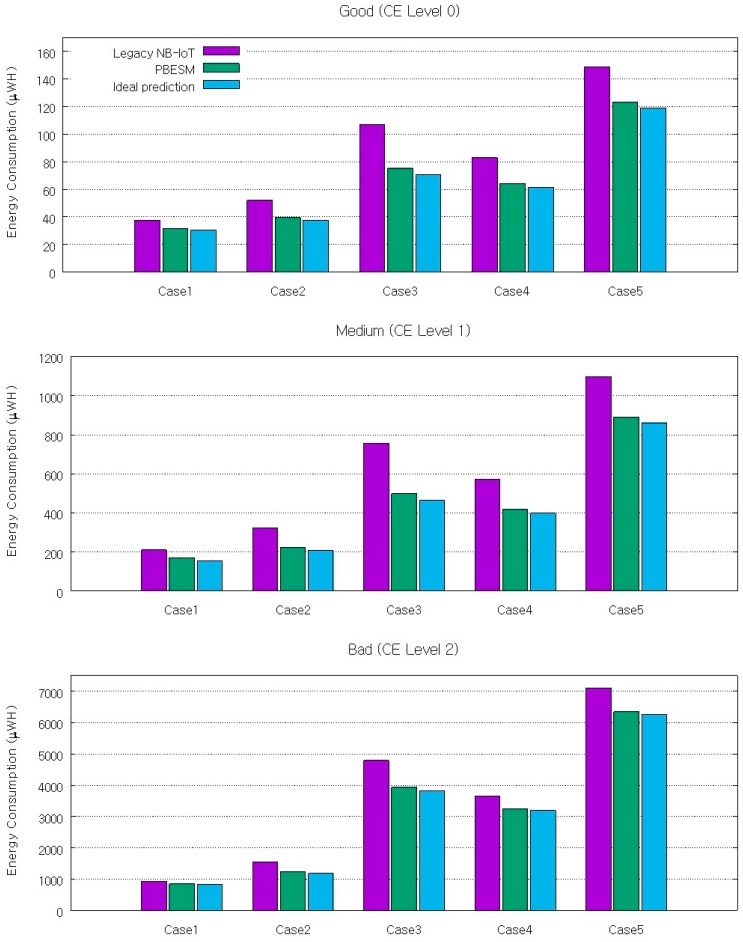
Energy consumption according to channel condition for legacy NB-IoT, PBESM, and ideal prediction.

For energy consumption, the PBESM outperforms the legacy NB-IoT from 10% to 34% regardless of the channel quality, as shown in Figure 15. We can see that there is a slight decline in performance from 3% to 5% compared to the ideal prediction. Given that the uplink transmission is delayed by the prediction error, the total active time for one session can increase, but the radio transmission time and energy consumption decrease. Hence, the proposed scheme is effective in terms of energy consumption. For instance, when we assume a scenario of a metering device with a 5 Wh AA battery and check the energy consumption everyday through the network, the battery life is 12.26 years for case 4 and medium channel quality in the legacy IoT, but it increases to 15.95 years by applying the proposed mechanism.

**Figure 16:**
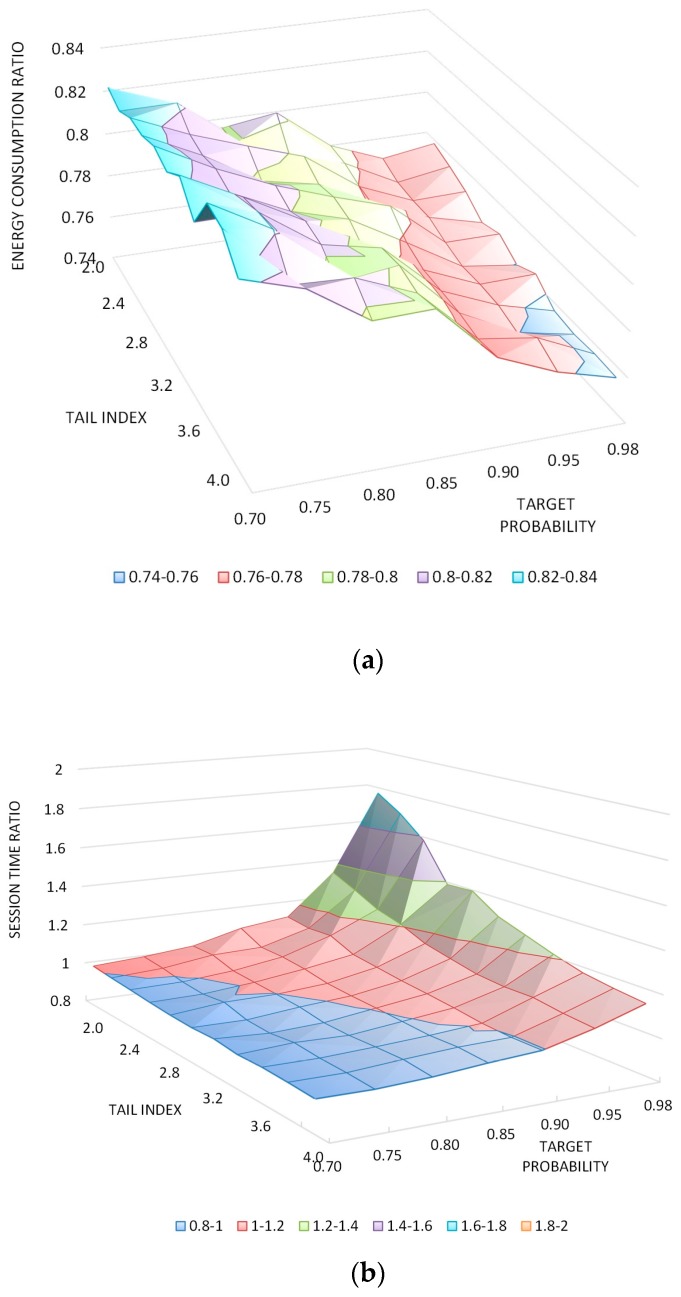
Ratio of PBESM to legacy NB-IoT according to tail index and target probability: (**a**) energy consumption; (**b**) active time.

Figure 16 shows the energy consumption and active time according to the processing delay distribution and target probability of successful delay prediction obtained from the PBESM algorithm. These results correspond to case 4 for a good channel quality. A high target probability of successful delay prediction in the PPE results in energy saving across more sessions. However, the prediction error also increases and time to complete the transmission becomes excessively larger than that for the legacy NB-IoT. In real environments, the target probability should consider the quality-of-service deadline of the session. For medium and bad channel quality, we recommend using 90% as the target probability. Considering the processing delay distribution, as the tail index becomes larger, the processing delay can be predicted with a smaller error; thus, it can improve both active time and energy consumption. 

### 5.4. Prediction of Processing Delay

The energy consumption using the PBESM ($E_{PBESM}^{\left(i,j\right)}$) should be smaller than that using the legacy NB-IoT procedure ($E_{legacy}^{\left(i,j\right)}$), where i is the CID and j is the PID. The energy consumed by the legacy NB-IoT is

(3) $$\begin{array}{ll} E_{legacy}^{\left(i,j\right)} & ≅\left(T_{NPRACH}^{\left(i\right)}+T_{MSG3}^{\left(i,j\right)}+T_{NPUSCH}^{\left(i,j\right)}\right)\cdot PW_{TX}^{\left(i\right)}\\ & +\left(T_{NPDCCH}^{\left(i\right)}+T_{RAR}^{\left(i\right)}+T_{NPDCCH}^{\left(i\right)}\right)\cdot PW_{RX}^{\left(i\right)}+T_{GAP}^{\left(i\right)}\cdot PW_{sleep}^{\left(i\right)} \end{array}$$

where $T_{X}$ is the execution time for procedure *X* including retransmission, $PW_{X}$ is the required power for procedure *X*, *RAR* is random-access-response procedure and *GAP* is the time gap between *NPRACH* and *RAR*. In Equation (3), we assume that the energy by waiting for an NPRACH opportunity is negligible. On the other hand, the energy consumed using the proposed scheme is

(4) $$E_{PBESM}^{\left(i,j\right)}=P_{o}^{\left(i,j\right)}\cdot (T_{o}^{\left(i,j\right)}\cdot PW_{sleep}^{\left(i\right)}+T_{NPUSCH}^{\left(i,j\right)}\cdot PW_{TX}^{\left(i\right)})+\left(1-P_{o}^{\left(i,j\right)}\right)\cdot E_{legacy}^{\left(i,j\right)}$$

where $P_{o}$ is the target probability of successful delay prediction and $T_{o}$ is the predicted processing delay. When the prediction succeeds, it is possible to save energy from the random-access procedure; otherwise, it uses same energy as the legacy NB-IoT procedure. Considering $E_{legacy}^{\left(i,j\right)}\geq E_{PBESM}^{\left(i,j\right)}$, Equations (3) and (4) become

(5) $$T_{o}^{\left(i,j\right)}\leq T_{NPRACH-GAP}^{\left(i\right)}+\left(2\cdot T_{NPDCCH}^{\left(i\right)}+T_{RAR}^{\left(i\right)}\right)\cdot PW_{RX^{\prime }}^{\left(i\right)}+\left(T_{NPRACH}^{\left(i\right)}+T_{MSG3}^{\left(i,j\right)}\right)\cdot PW_{TX\prime }^{\left(i\right)}$$

where the power ratios with respect to the sleep power are $PW_{RX^{\prime }}^{\left(i\right)}=PW_{RX}^{\left(i\right)}/PW_{sleep}^{\left(i\right)}$ and $PW_{TX^{\prime }}^{\left(i\right)}=PW_{TX}^{\left(i\right)}/PW_{sleep}^{\left(i\right)}$. For example, $PW_{RX^{\prime }}^{\left(i\right)}=\frac{60\;\mathrm{mW}}{3\;\mathrm{mW}}=20$ and $PW_{TX^{\prime }}^{\left(i\right)}=\frac{460\;\mathrm{mW}}{3\;\mathrm{mW}}=153.3$ for the metrics used in the simulation. Equation (5) shows the upper boundary of the predicted processing delay, and we notice that a high ratio of transmission to sleep power allows a wide margin for the prediction error.

### 5.5. Probability of Successful Prediction

When the prediction entities fail to predict the processing delay or uplink occurrence, the UE avoids transmitting an empty packet at the prescheduled time, thus preventing battery consumption. However, the radio resources reserved for NPUSCH are not used and reduce the cell capacity. To generalize the unused radio resource, we compare the radio resources of the legacy NB-IoT procedure with those of the proposed PBESM:

(6) $$P_{ul}^{\left(i,j\right)}\cdot N_{legacy}^{\left(i,j\right)}\geq P_{ul}^{\left(i,j\right)}\left\{P_{s}^{\left(i,j\right)}\cdot N_{PBESM}^{\left(i,j\right)}+\left(1-P_{s}^{\left(i,j\right)}\right)N_{legacy}^{\left(i,j\right)}\right\}+\left(1-P_{ul}^{\left(i,j\right)}\right)\left(1-P_{s}^{\left(i,j\right)}\right)\cdot N_{NPUSCH}^{\left(i,j\right)}$$

where:

- $P_{s}^{\left(i,j\right)}$ is the target probability of successful prediction for the uplink packet occurrence,
- $P_{ul}^{\left(i,j\right)}$ is the probability of uplink packet occurrence to the downlink packet in the NB-IoT device, and
- $N_{X}^{\left(i,j\right)}$ the number of the radio resource units for procedure *X* including repeating transmission.

The number of radio resources used in the legacy NB-IoT and proposed scheme are, respectively,

(7) $$N_{legacy}^{\left(i,j\right)}=N_{NPRACH}^{\left(i\right)}+N_{NPDCCH\_RAR}^{\left(i\right)}+N_{NPDSCH\_RAR}^{\left(i\right)}+N_{NPUSCH\_MSG3}^{\left(i,j\right)}+N_{NPDCCH}^{\left(i,j\right)}+\;N_{NPUSCH}^{\left(i,j\right)}$$

(8) $$N_{PBESM}^{\left(i,j\right)}=P_{o}^{\left(i,j\right)}\cdot N_{NPUSCH}^{\left(i,j\right)}+\left(1-P_{o}^{\left(i,j\right)}\right)\cdot (N_{NPUSCH}^{\left(i,j\right)}+N_{legacy}^{\left(i,j\right)}).$$

Then, Equation (6) becomes

(9) $$P_{o}^{\left(i,j\right)}\geq \frac{N_{\mathrm{NPUSCH}}^{\left(i,j\right)}}{N_{\mathrm{legacy}}^{\left(i,j\right)}}\times \varphi^{\left(i,j\right)}$$

where $\;\varphi^{\left(i,j\right)}=\left(1-\frac{1}{P_{ul}^{\left(i,j\right)}}\right)\cdot \left(1-\frac{1}{P_{s}^{\left(i,j\right)}}\right)+1\geq 1$.

Equation (9) shows that the lower boundary for the target probability is proportional to the ratio between the number of resources for NPUSCH and the legacy procedure. In addition, the target probability should increase with the uplink packet size.

## 6. Conclusions

In this paper, we introduced the energy consumption problem caused by the scheduling request procedure in the NB-IoT network. NB-IoT characteristics such as repeating transmission of NPUSCH and NPRACH, increase energy consumption in an IoT device, thus notably decreasing the battery life. As a solution, we propose the PBESM to reduce energy consumption by decreasing the number of scheduling request procedures. The PBESM predicts the uplink occurrence through inspection of the packet header and classification of the predictable message. Furthermore, it measures the response time for each session and predicts the processing delay following the proposed algorithm. A network-level simulation showed that the PBESM can achieve from 10% to 34% battery saving in different scenarios and improve the total active time per session by up to 16%, if compared to the legacy NB-IoT. Even in the worst case that corresponds to a very short time session with good channel quality, the active time increased to some extent, but the energy consumption was dramatically reduced. 

Further works will include a software-defined network architecture to enhance packet inspection. Moreover, we will investigate the effect of contention resolution in a multiuser scenario. We expect that the failure of contention resolution will decrease and the PBESM will raise the cell capacity.

Overall, the proposed mechanism does not require any special hardware on an IoT device, but a simple modification in the network entities including packet inspection and delay prediction. We believe that our mechanism can contribute to successful deployment of NB-IoT networks.

## Figures and Tables

**Table 1:** Predictable packet types.

Packet Type	Prediction Entity	Strategy
RLC protocol	PPE	Prediction of RLC ACK packet though poll bit transmission
RRC protocol	PPE	Prediction of RRC response message through a REQUEST message type
NAS protocol	PIE	Prediction of NAS response message through a REQUEST message type
Transport layer protocol	PIE	Prediction for DNS, DTLS, etc. through packet header inspection
Application response	Application Server, PIE	Application server can analyze application message type

**Table 2:** Simulation parameters.

Parameter	Value
ul_K0_for_DCI_formatN0	8 ms
ul_K0_for_DCI_formatN0_for_random-access response	12 ms
dl_K0_for_DCI_formatN1	0
k0_for_ack_nack_pusch	13
NPRACH cyclic prefix0	267 µs
Number of uplink subcarrier	6, fixed
Scheduling size for MSG3	88 bits

**Table 3:** Parameters for Each Channel Quality According to MCL.

Parameter	Good (144 dB)	Medium (154 dB)	Bad (164 dB)
NPRACH periodicity	80 ms	640 ms	1280 ms
Number of repetitions for NPDCCH	4	32	64
NPDCCH StartSF CSS for USS	2	16	32
Number of repetition for NPUSCH	2	16	32
Number of repetition for NPDSCH	16	128	256
Number of repetitions for HARQ ACK/NACK	8	64	128
Number of repetitions per preamble attempt	8	64	128
NPDCCH number of repetitions for random access	4	32	64
NPDCCH StartSF CSS for random access	2	6	32
NPUSCH carrier spacing	15 kHz Multitone	15 kHz Multitone	3.75 kHz Simgle-tone

**Table 4:** Scenarios.

Scenario	Case 1	Case 2	Case 3	Case 4	Case 5
UE report scenario	X				
Network command scenario		X	X	X	X
Non-IP	X	X			
IP			X	X	X
CoAP/DTLS/UDP			X	X	
Initial security context creation			X		
Resuming security context				X	
TCP					X

**Table 5:** Power Consumption According to UE State.

State	Power	Assumption
TX	460 mW	23 dBm (50% power amplifier efficiency) and 60 mW (other circuitry)
RX	60 mW	Includes digital mixing/decimation and demodulation
Sleep	3 mW	Maintains accurate timing (keeping RFs)
Deep sleep	0.016 mW	Common assumption (RTC)
